# Decoding the Role of DVL1 in Intracranial Meningioma

**DOI:** 10.3390/ijms222111996

**Published:** 2021-11-05

**Authors:** Anja Bukovac, Katarina Dragičević, Anja Kafka, Darko Orešković, Sanja Cesarec-Augustinović, Nives Pećina-Šlaus

**Affiliations:** 1 Laboratory of Neurooncology, Croatian Institute for Brain Research, School of Medicine, University of Zagreb, 10000 Zagreb, Croatia; anja.kafka@mef.hr (A.K.); nina@mef.hr (N.P.-Š.); 2 Department of Biology, School of Medicine, University of Zagreb, 10000 Zagreb, Croatia; 3 Biotech Research & Innovation Centre, University of Copenhagen, DK-2200 Copenhagen, Denmark; katarina.dragicevic@bric.ku.dk; 4 Department of Neurosurgery, University Hospital Dubrava, 10000 Zagreb, Croatia; darkoreskov@gmail.com; 5 “Ljudevit Jurak” Department of Pathology and Cytology, Clinical Hospital Center “Sestre milosrdnice”, 10000 Zagreb, Croatia; sanja.cesarec@kbcsm.hr

**Keywords:** DVL1, PDZ domain, β-catenin, Wnt signaling pathway, intracranial meningioma

## Abstract

In the search for molecular candidates for targeted meningioma therapies, increasing attention has been paid to the role of signaling pathways in the development and progression of intracranial meningiomas. Although it is well known that the Wnt signaling pathway is involved in meningioma progression, the role of its central mediator, DVL1, is still unclear. In order to investigate the influence of *DVL1* gene alterations on the progression of human intracranial meningioma, we focused on its central PDZ domain, which is responsible for DVL interaction with the Fzd receptor and the phosphorylation of DVL mediated through the casein kinases CK1 and CK2. A genetic analysis of genomic instability revealed the existence of microsatellite instability in 9.09% and the loss of heterozygosity in 6.06% of the samples. The sequencing of the PDZ gene region showed repetitive deletions of two bases located in intron 7 and exon 8, and a duplication in intron 8 in most samples, with different outcomes on the biological function of the DVL1 protein. Immunohistochemistry revealed that the nuclear expression of DVL1 was significantly correlated with a higher expression of active β-catenin (*p* = 0.029) and a higher meningioma grade (*p* = 0.030), which leads to the conclusion that it could be used as biomarker for meningioma progression and the activation of the Wnt signaling pathway.

## 1. Introduction

Intracranial meningioma, with their mostly benign nature and slow growth, can still progress and show malignant characteristics that can lead to poor outcome for patients. According to statistics from the latest CBTRUS report (The Central Brain Tumor Registry of the United States) from 2012 to 2016 [1], the percentage of meningioma in the total sample of brain tumors is 37.1%, and among all benign brain tumors it is at 53.1%. The abovementioned numbers suggest that meningiomas are currently the most common primary tumors of the central nervous system [2], making the discovery of mechanisms involved in their development and progression highly valuable.

Molecular mechanisms underlying meningioma progression are usually connected to aberrant signaling pathways such as PI3K-AKT-mTOR, Ras-Raf-MEK, Rac-PAK-JNK, TGFβ-SMAD, RB, p53, Hedgehog, Notch and canonical Wnt [3,4,5,6,7,8,9]. This study concentrates on the canonical Wnt pathway, whose regulation of cell growth, development, and survival has great importance in tumorigenesis.

In tumors, Wnt signaling can play different roles; for instance, allowing them to reprogram their metabolism or promote chronic inflammation and oxidative stress, or enabling resistance to immunotherapy [10]. It has been shown that the activation of Wnt signaling can induce the malignant transformation of neural stem cells and thus contribute to the development of primary brain tumors [11,12,13,14,15,16]. The Wnt signaling pathway in meningiomas is known to be activated and plays a role in progression [17,18]. Activation occurs when Wnt ligands bind to membrane receptors called Frizzled. This binding is followed by the activation of a specific member of the Disheveled (Dvl) protein family, which is then pulled to the membrane where it promotes the phosphorylation and disassembly of the destruction complex, which targets β-catenin. The destruction of the central oncogenic molecule β-catenin is thus impaired, leading to its accumulation in the cytoplasm, followed by the translocation to the nucleus where it binds to the TCF/LEF factors and stimulates the transcription of other targeted oncogenes [19,20,21,22]. It has also been shown that DVL has nuclear activity by binding to the TCF factor together with the β-catenin and by activating the transcription of Wnt target genes [23].

The Disheveled family is a highly conserved, multifunctional group of proteins with three human homologues—DVL1, DVL2, and DVL3. The excessive expression of Disheveled proteins has been proven to cause increased activation of Wnt signaling in many different types of tumors [19]. All members of the DVL protein family contain three basic conserved domains—DIX, DEP, and PDZ. On the amino terminal side resides the DIX domain, which represents an important binding site for the AXIN [23]. On the carboxyl terminal side of the DVL protein is the DEP domain, which is key in DVL protein interactions with DAAM1 (Disheveled associated activator of morphogenesis 1) [23]. Between these two domains lies the central PDZ domain (Postsynaptic Density 95, Discs Large, Zonula Occludens-1) composed of 73 amino acids. The PDZ domain plays a role in both the canonical and non-canonical form of Wnt signaling and has the utmost importance in mediating the different interactions that DVL proteins carry out. The PDZ domain is essential for DVL interaction with the carboxyl terminal domain of the Fzd receptor, and also plays a role in DVL phosphorylation mediated through the casein kinases CK1 and CK2 [23].

The aim of this study was to determine genetic alterations in the *DVL1* gene and its central PDZ domain, and their role in the progression of intracranial meningioma. The results of this study could contribute to the discovery of new prognostic biomarkers and targets for personalized therapies.

## 2. Results

Out of 33 samples of intracranial meningiomas, 22 samples (66.67%) belonged to female and 11 samples (33.33%) to male patients. The age of the patients ranged from 23 to 85 years, with a mean of 61.12 and a median of 67 years.

Moreover, out of 33 samples, 16 were classified as grade I, 12 as grade II, and 5 as grade III. Using the Kruskal–Wallis test, there was no statistically significant difference between the incidence of grade with respect to the age of the patients (*p* = 0.259). Using the Fisher test, a statistically significant difference was found between the grade and sex of the patients (*p* = 0.026), where men showed a greater tendency to develop higher grade meningiomas and women the lower ones.

### 2.1. Genetic Instability of the DVL1 Gene Recorded with the D1S468 Marker

**Figure 1:**
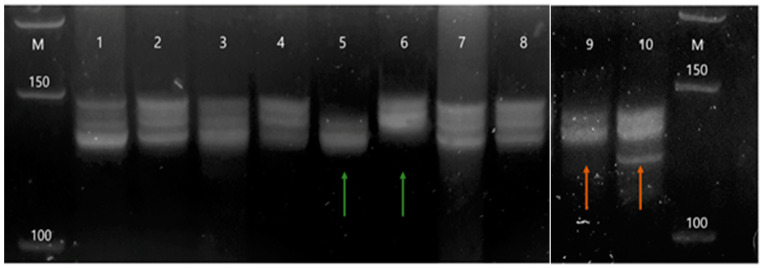
Examples of genetic changes—MSI and LOH, found in intracranial meningioma and detected on Spreadex gels. Legend: M—marker; odd numbers—tumor samples; even numbers—blood samples; green arrows—example of MSI in a tumor sample; red arrows—example of LOH in a tumor sample.

The *DVL1* microsatellite marker D1S468 proved to be highly informative, with a heterozygosity present in all samples. Out of 33 intracranial meningiomas analyzed, three samples (9.09%) showed microsatellite instability (MSI) and two samples (6.06%) demonstrated the loss of heterozygosity (LOH). The total number of samples in which the *DVL1* gene was altered was 15.15%. Out of the five samples in which one of these changes was found, four belonged to female patients. Based on the results of the Fisher test, no statistically significant difference was found between the sex and the genetic alteration (ρ = 1.000, *p* = 0.542), nor between the grade and the genetic alteration (ρ = 0.053, *p* = 1.000). On the contrary, Pearson’s test showed a statistically significant difference between the incidence of genetic alteration and patients’ age (r = −0.485, *p* = 0.004), indicating that younger patients harbored more alterations. Examples of MSI and LOH changes are shown in Figure 1.

### 2.2. Mutations in the PDZ Region of the DVL1 Gene

**Figure 2:**
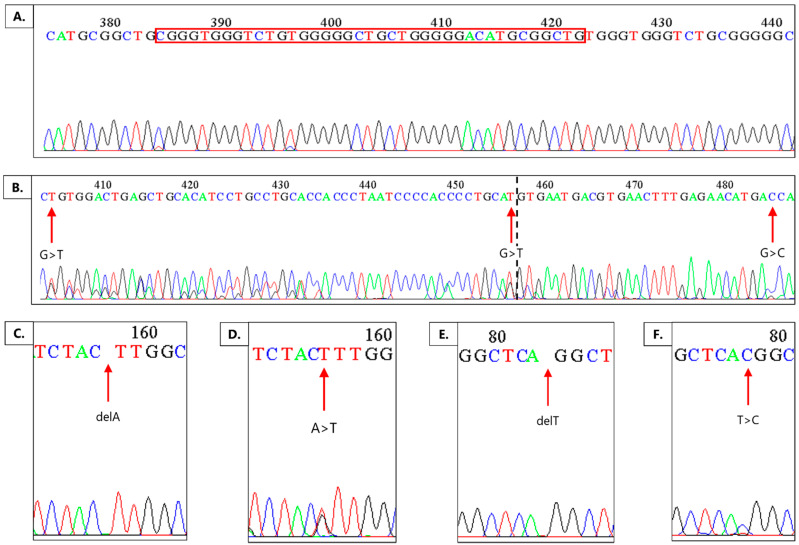
Mutations detected by the Sanger sequencing method. (**A**)—g.14228_14267dup (red frame); (**B**)—g.14248G>T, g.14300G>T, g.14329G>C; (**C**)—g.14004delA; (**D**)—g.14004A>T; (**E**)—g.13921delT; (**F**)—g.13921T>C. The dashed line indicates the pre-mRNA splicing site.

Out of a total of 33 samples, the high-resolution melting method (HRM) revealed observable curve deviations in most samples; out of these, 10 samples (30.30%) showed large deviations of the tumor curve from the control blood curve. A total of 26 samples of tumor DNA were available for Sanger sequencing. A comparison of tumor sequences with the reference sequence revealed the existence of nucleotide changes in all of the sequenced samples, which are systematized in Table 1 and shown in Figure 2.

Altogether, nine different types of mutations were found (Table 1). Twelve patients harbored one mutation, seven had two mutations, five had three mutations, one patient harbored four mutations, and another one had five different mutations. The mutation with the highest frequency was the g.14228_14267dup found in 25 samples (96.15%), followed by the deletion g.14004delA found in nine samples (34.61%) and g.13921delT found in seven samples (26.92%). The most pronounced mutation was the intron 8 duplication, which is 39 bp long (g.14228_14267dup), as shown in Figure 2a. This duplication was not registered in the reference sequence available at the National Center for Biotechnology Information (NCBI) database, but was found in 25 out of our 26 analyzed samples. The effect of the duplication on the biological function of the DVL1 protein could not be determined by the PROVEAN program.

Intron 7 showed a particular location, g.13921, where two different mutations occurred in 11 samples—the deletion of nucleotide T (g.13921delT) (Figure 2e) and the substitution of the same nucleotide T into C (g.13921T>C) (Figure 2f). The effect of both mutations on the biological function of the DVL1 protein was not determined by the PROVEAN program. Our further analysis showed that both mutations were linked to the lower expression and H-score value of the DVL1 protein (r = −0.478, *p* = 0.038). Furthermore, the deletion of nucleotide T was significantly more tied to lower grade than to the higher grades (χ = −0.434, *p* = 0.027), with no incidence observed in malignant grades. On the other hand, the substitution of the same nucleotide, was mostly present in malignant grades and a significant correlation to higher grades was established (χ = 0.514, *p* = 0.032). Moreover, the deletion of T was found exclusively in female patients (χ = −0.442, *p* = 0.024).

Exon 8 showed two locations hit by three different mutations in 11 samples. Two deletions of nucleotide A in exon 8 (g.13998delA and g.14004delA) caused harmful frameshift mutations. The more frequent deletion of nucleotide A (g.14004delA) (Figure 2c) is located at the codon ATT, which codes for the amino acid isoleucine. The change in the reading frame caused by this deletion introduced a downstream stop codon and caused the formation of the truncated DVL1 protein, with altered activity and functionality. The substitution of nucleotide A into T (g.14004A>T) (Figure 2d) at the same location caused the codon change from ACC to TCC, and consequently the amino acid change from Ile to Ser. Using a PROVEAN tool to analyze the effect of protein variations, this mutation was flagged as harmful for the DVL1 function. This substitution was found in a single sample that belonged to the meningothelial subtype of grade I meningioma. However, this sample showed the highest expression of active β-catenin (H-score = 262) and the highest percentage of nuclei with the DVL1 expression (90%).

The only sample that did not harbor the aforementioned duplication had two mutations in the intron 8—g.14248G>T and g.14300G>T. Mutation g.14300G>T is located at the splicing site between intron 8 and exon 9, and g.14248G>T is also located nearby. Due to their locations, these changes are potential splice site mutations that may change the length of the DVL1 protein (Figure 2b).

In exon 9, the substitution of g.14329G>C (Figure 2b) caused a change in the amino acid Ser to Thr. However, the analysis by PROVEAN showed that this change caused no disruption to the tertiary and quaternary structures of the DVL1 protein, nor a change in its biological function.

### 2.3. Protein Expression and Localization of the DVL1 and Active β-Catenin Form

**Figure 3:**
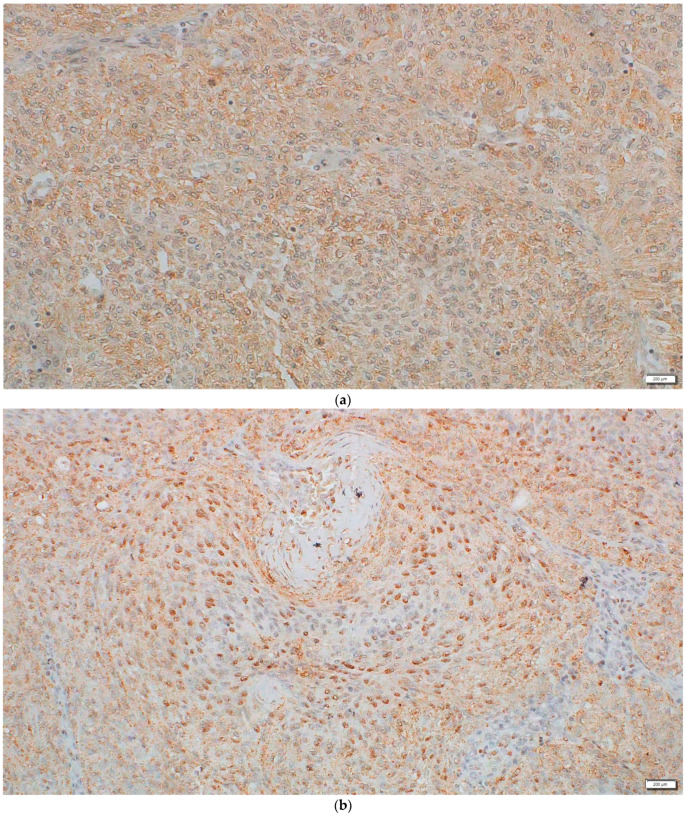
(**a**) Cytoplasmic localization of DVL1 in intracranial meningioma, with moderate to high expression. The specimen was taken from a 70-year-old female patient who was diagnosed with atypical (grade II) meningioma. (**b**) Nuclear localization of DVL1 in intracranial meningioma, with high expression. The specimen was taken from a 58-year-old female patient who was diagnosed with atypical (grade II) meningioma. Magnification: 200×, scale bar: 200 μm.

The protein expression evaluation using the H-score revealed the presence of DVL1 in all inspected samples, mostly localized in cytoplasm but also in the nuclei (Figure 3). The H-score mean for DVL1 expression was 169.74. The majority of samples (16/23) showed a moderate signal (70%), while six samples showed a strong signal (26%). One sample did not show cytoplasmic expression but had a pronounced nuclear expression. A total of 48% of the samples did not express DVL1 in the nuclei, or expression was in less than 5% of the nuclei in the field of view. In 7/23 samples (30%), DVL1 was expressed in 10–50% of nuclei, while 5/23 samples (22%) showed the frequent nuclear expression of DVL1 in more than 50% of nuclei in the field of view. Spearman′s test revealed that DVL1 H-score values dropped with the age of the patients (ρ = −0.752, *p* = 0.000). Moreover, the highest values of the DVL1 H-score (>200) were correlated with samples without any mutation in the PDZ domain (r = −0.517, *p* = 0.023), while samples comprised of mutations in the PDZ domain expressed less DVL1 protein product. The duplication g.14228_14267dup was omitted from calculation since it appeared in 96% of the analyzed meningiomas. Furthermore, the nuclear expression of the DVL1 protein was significantly correlated with the higher grade (ρ = 0.453, *p* = 0.030) and expression of the active form of β-catenin (ρ = 0.456, *p* = 0.029). On the contrary, Pearson′s test showed no significant correlation between active β-catenin and cytoplasmic DVL1 H-score values (r = 0.101, *p* = 0.647).

Active or non-phosphorylated β-catenin was present in all samples and was localized in the cytoplasm of tumor cells. The mean H-score value of all samples was 100.17. The most pronounced was the low expression of active β-catenin, which was observed in 12/23 samples (52%). In 9/23 samples (39%) active beta-catenin had a moderate signal, and two samples (9%) showed a strong signal. No nuclear expression was found. Higher expressions of active β-catenin were significantly correlated with higher grades (ρ = 0.580, *p* = 0.004).

## 3. Discussion

Our findings concerning alterations of the *DVL1* gene in the intracranial meningioma set showed that 15.15% of the samples harbored changes. Microsatellite instability was present in 9.09% of the samples, and the loss of heterozygosity was observed in 6.06%. The genomic changes were not significantly associated with tumor grade or the sex of the patients. However, MSI was most frequently found in grade III and may be linked to the rise of mutational burden. On the contrary, genetic alterations were associated with patients younger than 60 years (r = −0.485, *p* = 0.004). When considering LOH, similar findings were described by Nagahata et al. [24], who studied *DVL1* in breast cancer and found that 10% of the samples harbored LOH of the *DVL1* gene, suggesting that *DVL1* has a role in breast cancer progression. Our previous work on *DVL1* in astrocytoma [25] showed the high frequency of MSI in all grades of astrocytomas, while LOH was detected only in glioblastomas (in 8.6% of samples). Our present results showed that the LOH of *DVL1* was not tied to the malignant grade and may represent early event in meningioma progression. Kafka et al. [25] investigated two different microsatellite markers, one of which was D1S468 and demonstrated higher frequency of genomic instability. Therefore, in future meningioma studies, samples could be tested with two *DVL1* gene microsatellite markers to show if a higher frequency of genomic instability is present.

A more detailed genetic analysis of the functionally crucial PDZ domain using Sanger sequencing showed a high mutation rate in the investigated sample. The most prominent was the duplication in intron 8, g.14228_14267dup, for which predictive software could not determine a significant impact on the DVL1 protein. Since the duplication was also found in the blood sequence of some patients, this may suggest a potential polymorphism or the germline existence of this variation. Such a 39 bp long duplication located within the intronic sequence may affect splicing signals. Many studies have shown that indel and other point mutations in introns, as well as changes at splicing sites, may all have an impact on proper splicing mechanisms [26,27,28,29]. Therefore, a duplication of 39 bp could lead to changes in secondary pre-mRNA structures affecting proper splicing. We can theorize that the new secondary structure causes the retention of the existing intron, resulting in a longer protein. On the other hand, duplication can cause the RNA loop that consists of an intron, but also includes one of the adjacent exons, resulting in a shorter protein. As described in a paper written by Lin et al. [30], structural stems at splicing sites can cause the formation of mRNA isoforms specific to some diseases, often tumors. The stem structures can also sterically impair the binding of splicing enhancers. It has also been shown that the higher percentage of GC base pairs positively correlates with the percentage of alternatively spliced exons. This fact is especially interesting in the context of studying the PDZ domain because in this region, a high percentage of GC base pairs naturally occurs, which is further increased by the duplication g.14228_14267dup riddled with GC.

The effect of two mutations at the same locus in intron 7, g.13921delT and g.13921T>C, were not determined by the PROVEAN program. Our study revealed that mutations at these loci were linked to the lower expression of the DVL1 protein (r = −0.478, *p* = 0.038), with the deletion of nucleotide T more tied to the lower grade (χ = −0.434, *p* = 0.027), no incidence in the malignant grades, and the substitution of the same nucleotide more frequent in the highest grade (χ = 0.514, *p* = 0.032). Likewise, the deletion of T was found exclusively in female patients (χ = −0.442, *p* = 0.024). These results prove that mutations at locus g.13921 downregulate DVL1 expression, and depending on the type of mutation, this effect will manifest in different grades. Additionally, the substitution of T (g.13921T>C) could be a marker of low DVL1 expression in higher grade meningioma patients.

The mutation with the highest potential effect is the deletion of nucleotide A in exon 8, g.14004delA. The analysis found that this mutation causes a frameshift and the consequent truncated version of the DVL1 protein. Such a premature stop codon in exon 8 of the region encoding the PDZ domain may act in favor of tumor formation and development. In their study, Brennan et al. [31] found that truncated versions of the LRP5/6 co-receptor, involved in the Wnt signaling pathway, may have a protooncogenic role. Truncated forms of the co-receptor stabilize β-catenin independently of other membrane proteins involved in Wnt signal transduction, and are resistant to degradation and endocytosis. In addition to LRP5/6, the role of the truncated form of the APC protein in colorectal tumor was also established by Schneikert and Behrens [32]. According to their research, the truncated form of the APC protein stimulates the migration of colorectal tumor cells and promotes the development of chromosomal instability. These studies suggest that the truncated form of the DVL1 protein caused by the g.14004delA mutation in 34.61% of samples could be characteristic of meningiomas and enhance Wnt signaling, thus stimulating tumorigenesis. The substitution at the same locus (g.14004A>T) was associated with the highest expression of active β-catenin (H-score = 262) and the highest percentage of nuclear DVL1 expression (90%). These findings are also conclusive with regard to the activation of Wnt signaling. Since it was found in only one sample, this hypothesis should be tested with a higher number of tumor samples harboring this mutation.

The only sample that did not harbor the duplication showed five other different mutations, three of which were the substitution of nucleotide G. The substitutions g.14248G>T and g.14300G>T in intron 8 were assigned as mutations of the splicing site. Due to these two substitutions, a longer DVL1 protein retaining part of intron 8 is possible. The third substitution, g.14329G>C, causes the change from amino acid Ser to Thr, which have similar biochemical properties and therefore probably have no impact on the structure and function of the DVL1 protein, as stated in the paper by Castro-Chavez [33]. However, if there is a change at the splicing site due to the first two mutations in intron 8, there is a possibility of a new reading frame that affects the substitution in exon 9, leading to the formation of a protein with an altered amino acid composition and a new structure.

Although the PDZ domain was severely mutated, the protein expression of DVL1 was found in all samples, mostly with a moderate signal (70%). However, a strong signal was less present in 26% of the samples, while one sample showed a lack of cytoplasmic expression but had a pronounced nuclear expression. Furthermore, we established that the highest values of the DVL1 H-score (>200) were correlated with samples without any mutation in the PDZ domain (r = −0.517, *p* = 0.023), indicating that samples containing mutations in the PDZ domain expressed significantly less DVL1 product (the duplication g.14228_14267dup was omitted from the calculation). In 48% of the analyzed meningiomas, the nuclear expression of DVL1 was missing; however, 22% of the samples still showed DVL1 expression in more than 50% of the nuclei. Although studies rarely report on the nuclear expression of DVL1 in different tumors [34,35], Sharma et al. [36] demonstrated that the acetylation of the conserved lysines (K69 and K285), which are present in the DIX and PDZ domains, not only promoted the nuclear localization of DVL1, but also influenced its promoter binding and the regulation of genes implicated in cancer. Furthermore, our study showed that the cytoplasmic expression of DVL1 was not correlated with meningioma grade or the expression of active cytoplasmic β-catenin (r = 0.101, *p* = 0.647). However, this was not the case with nuclear DVL1 expression, which was associated with higher grades (ρ = 0.453, *p* = 0.030) and higher expressions of β-catenin′s active form (ρ = 0.456, *p* = 0.029). This may suggest that the nuclear expression of DVL1 could promote Wnt signaling activation and potentially serve as a biomarker of meningioma progression. Similar findings on the influence of DVL1 progression in other tumors were also reported. For instance, in a study by Karin-Kujundzic et al. [37], the active involvement of DVL1 and significantly higher DVL1 expressions in serous ovarian carcinomas as compared to normal ovarian tissue were reported. Mizutani et al. [38] found that expressions of DVL1 and β-catenin are correlated, and that DVL1 expression increases with grade in prostate cancer. On the other hand, Ameli et al. [35] concluded that in invasive ductal and lobular breast carcinoma, DVL1 does not correlate with grade; however, they did not find a nuclear expression of the protein. Wei et al. [39] showed that the expression levels of DVL1 were higher in non-small-cell lung cancer metastases and correlated to β-catenin expression, while Zhang et al. [40] demonstrated that DVL1 increases the accumulation of β-catenin in ovarian cancer cells. They also showed that DVL1 was responsible for the nuclear translocation of β-catenin, which was not compatible with our study. However, similar findings to ours were recorded by the Kafka et al. [41]. They showed that most brain metastases (45.2%) had moderate DVL1 expression levels, with nuclear staining in 54.8% of the cases. In addition, they reported a correlation between the nuclear expression of β-catenin and upregulated DVL1 expression.

Interestingly, in our study, DVL1 H-score values dropped with the age of the patient (ρ = −0.752, *p* = 0.000). This finding is consistent with our previous study on the involvement of the Disheveled protein family in astrocytoma malignancy grades [25], which showed that younger patients had a stronger DVL1 expression than the older ones. The same study indicated that high-grade tumors had a lower expression of DVL1, suggesting that it may be an early event, which is contrary to our present findings on meningioma.

Active β-catenin, which is distinctive for activated Wnt signaling in tumor cells, was present in all samples and was localized in cytoplasm. The mean H-score value was 100.17. Low expression levels of active β-catenin were observed in 52% of meningiomas, while in 39% of the investigated samples of active β-catenin had a moderate signal, while two samples (9%) showed a strong signal. Although nuclear expression was not found, higher expression levels of active β-catenin were significantly correlated with higher grades (ρ = 0.580, *p* = 0.004) and nuclear DVL1 expression (ρ = 0.456, *p* = 0.029). This is in alignment with our previous study [18], indicating β-catenin involvement in meningioma progression.

## 4. Materials and Methods

Our study consisted of 33 samples of intracranial meningioma with different malignancy grades. All tumor samples were classified by pathologists according to the criteria of the World Health Organization [42]. Tumor samples, as well as 3–5 mL of autologous blood, were collected from patients with no prior radiation or chemotherapy treatments who were scheduled for operation. In addition to the pathohistological diagnosis and tumor grade, parameters such as the sex, location, and age of the patient were collected for each sample.

DNA was isolated from tumor tissue with the standard phenol/chloroform method [43], and from blood with the standard salting out method [44].

Samples were tested for genomic alternations—microsatellite instability (MSI) and loss of heterozygosity (LOH) of the *DVL1* gene by comparing DNAs from the tumor and blood of the same patient. Detection of MSI and LOH was performed on Spreadex gels EL400 Mini (Elchrom Scientific, AL-Labortechnik, AL-Diagnostic GmbH, Amstetten, Austria) using the microsatellite maker D1S468 (5′-TTAACCGTTTTGGTCCTACC-3′ and 5′-CTCTGACCAGCATTAAAGATTC-3′), with a high percentage of heterozygosity in the population.

For further investigation, to amplify the region encoding the PDZ domain, primers were designed using the NCBI database [45] and the primer design tool—Example Blast [46]. The primers (5′-TAACCGACTCCACCATGTCC-3′, 5′-GAAACGATCTCCCGCAGCA-3′) cover part of intron 7, whole exon 8 and intron 8, and part of exon 9 of the PDZ domain. Optimal PCR conditions for amplifications of the *DVL1* D1S468 microsatellite marker and the PDZ regions are shown in Table 2.

Mutations in the PDZ domain were detected using the high-resolution melting method (HRM) and LightCycler^®^ 480 High-Resolution Melting Master kit on the Roche LightCycler^®^ Nano System. By comparing DNA sequences from the tumor and blood of the same patient, potential mutations were revealed. All samples that were suspected to harbor mutation were sequenced using the standard Sanger sequencing method and BigDyeTerminator v3.1 Cycle Sequencing kit on ABI 3730XL (Applied Biosystems, Foster City, CA, USA). The obtained tumor sequences of the PDZ domain of the *DVL1* gene were compared with the sequence of the paired blood sample and with the sequence available at the NCBI database [47]. (Verification of the impact of detected mutations on the biological function of the DVL1 protein was performed using the publicly available PROVEAN tool [48] and immunohistochemistry.

Immunohistochemistry was performed on 4 µm thick paraffin-embedded sections of 23 available meningioma samples collected during surgery. The sections were collected during a period of four years. For DVL1 detection, we used a polyclonal rabbit anti-Disheveled/Dvl1 antibody: ab233003 (Abcam, Cambridge, UK), diluted 1:200; its recombinant fragment (His-T7-tag) corresponded to Human Dvl1 aa 150–300 and partly covered the central PDZ domain. We also tested the expression and localization of the active form of β-catenin using the monoclonal antibody non-Phospho beta-catenin (Ser33-37/Thr41) (D131A1) Rabbit mAb #8814 (Cell Signalling Technology, Danvers, MA, USA), diluted 1:800. For visualization, we used DAB chromogen (EnVision^TM^, Dako REAL^TM^). Slides with antibody-labelled tissue were analyzed using bright-field microscopy on the Olympus BX53 microscope. The expression of the protein was observed in tumor hot spots, where at least 200 cells were counted. Immunopositivity was quantified using the H-score (Equation (1)), with a range of protein expression values on a scale of 0–300 [49]:

H = [1 × (% of stations 1+) + 2 × (% of stations 2+) + 3 × (% of stations 3+)](1)

where 1+ indicates weak immunopositivity—yellowish / light brown color, 2+ indicates moderate immunopositivity—light brown, and 3+ indicates strong immunopositivity—dark brown. Depending on the obtained H-score value, samples were categorized into 3 groups: 0–100 = samples with no signal/weak signal (0/1+), 101–200 = samples with moderate signal (2+), and 201–300 = samples with strong signal (3+).

The results of the genetic and protein investigations were further analyzed and correlated using the publicly available program RStudio [50], including the R-package ggplot2, ggpubr, and plotly from the official R repository CRAN [51]. A significance level of *p* < 0.05 was used to process the results. The normality of the distribution was checked using the Shapiro–Wilk test.

## 5. Conclusions

We have shown that the central PDZ domain is highly mutated, with different outcomes on the biological function of the DVL1 protein. The samples containing mutations in the PDZ domain expressed significantly less DVL1 protein product, and the nuclear expression of DVL1 could potentially represent a good biomarker for meningioma progression and the activation of the Wnt signaling pathway. The results of this study contribute to a better understanding of the role of DVL1 in human intracranial meningiomas and point out molecules useful for diagnostics and the treatment of patients.

## Figures and Tables

**Table 1:** Types of mutations detected by Sanger sequencing.

Location of Mutation	Type of Mutation	Number of Samples with This Type of Mutation
Intron 7	NG_008048.2: g.13921delT	7
Intron 7	NG_008048.2: g.13921T>C	4
Exon 8	NG_008048.2: g.13998delA	1
Exon 8	NG_008048.2: g.14004delA	9
Exon 8	NG_008048.2: g.14004A>T	1
Intron 8	NG_008048.2: g.14228_14267dup	25
Intron 8	NG_008048.2: g.14248G>T	1
Intron 8	NG_008048.2: g.14300G>T	1
Exon 9	NG_008048.2: g.14329G>C	1

**Table 2:** PCR conditions for the *DVL1* microsatellite marker D1S468 and the PDZ genetic regions.

	(Pre) Denaturation	Denaturation	Annealing	Extending	No. of Cycles
D1S468	94 °C/5 min	94 °C/30 s	60 °C/30 s	72 °C/30 s	40
PDZ	94 °C/5 min	94 °C/35 s	58.6 °C/35 s	72 °C/35 s	35

## Data Availability

Data supporting the reported results are contained within the article. Some of the data presented in this study are available on request from the corresponding author. The data are not publicly available due to privacy issues.
